# Hyperglycemia Induces Skin Barrier Dysfunctions with Impairment of Epidermal Integrity in Non-Wounded Skin of Type 1 Diabetic Mice

**DOI:** 10.1371/journal.pone.0166215

**Published:** 2016-11-15

**Authors:** Junko Okano, Hideto Kojima, Miwako Katagi, Takahiko Nakagawa, Yuki Nakae, Tomoya Terashima, Takeshi Kurakane, Mamoru Kubota, Hiroshi Maegawa, Jun Udagawa

**Affiliations:** 1 Department of Anatomy and Cell Biology, Shiga University of Medical Science, Shiga, Japan; 2 Departments of Stem Cell Biology and Regenerative Medicine, Shiga University of Medical Science, Shiga, Japan; 3 Industry-Academia-Government Collaboration Center of Nara Medical University, Nara, Japan; 4 Internal Medicine, Shiga University of Medical Science, Shiga, Japan; "INSERM", FRANCE

## Abstract

Diabetes causes skin complications, including xerosis and foot ulcers. Ulcers complicated by infections exacerbate skin conditions, and in severe cases, limb/toe amputations are required to prevent the development of sepsis. Here, we hypothesize that hyperglycemia induces skin barrier dysfunction with alterations of epidermal integrity. The effects of hyperglycemia on the epidermis were examined in streptozotocin-induced diabetic mice with/without insulin therapy. The results showed that dye leakages were prominent, and transepidermal water loss after tape stripping was exacerbated in diabetic mice. These data indicate that hyperglycemia impaired skin barrier functions. Additionally, the distribution of the protein associated with the tight junction structure, tight junction protein-1 (ZO-1), was characterized by diffuse and significantly wider expression in the diabetic mice compared to that in the control mice. In turn, epidermal cell number was significantly reduced and basal cells were irregularly aligned with ultrastructural alterations in diabetic mice. In contrast, the number of corneocytes, namely, denucleated and terminally differentiated keratinocytes significantly increased, while their sensitivity to mechanical stress was enhanced in the diabetic mice. We found that cell proliferation was significantly decreased, while apoptotic cells were comparable in the skin of diabetic mice, compared to those in the control mice. In the epidermis, Keratin 5 and keratin 14 expressions were reduced, while keratin 10 and loricrin were ectopically induced in diabetic mice. These data suggest that hyperglycemia altered keratinocyte proliferation/differentiation. Finally, these phenotypes observed in diabetic mice were mitigated by insulin treatment. Reduction in basal cell number and perturbation of the proliferation/differentiation process could be the underlying mechanisms for impaired skin barrier functions in diabetic mice.

## Introduction

Skin complications are relatively common in diabetes. Xerosis (dry skin) is a skin disease characterized by persistent itchiness and fissures in diabetic patients, and it often delays the process of wound healing, resulting in reduction in the patients’ quality of life [1]. A foot ulcer is also a common skin disorder in diabetic patients. This skin damage becomes more critical when complicated by a bacterial infection [2]. In such a case, limb/toe amputation is often required to prevent the development of sepsis [3, 4].

Tragic outcomes from a limb/toe loss and higher possibilities of hospitalizations because of skin infections in diabetes [5] have increased the attention on study of non-wounded diabetic skin. However, the results are quite controversial. For instance, some researchers reported thinner epidermis in non-wounded skin in diabetes, while others determined no altered thickness of the epidermis in diabetes, compared with normal skin [6–10]. On the contrary, another study showed thicker epidermis in diabetes compared with normal skin [11]. Likewise, with respect to the molecular profile of non-wounded skin in diabetes, inconsistent results from studies using even the same type 2 diabetic mouse model, db/db mice, have been reported on commonly assessed skin proliferation/differentiation markers such as keratin 1 [8, 12]. A possible explanation would be that the alteration of such proteins depends on the age of db/db mice [13]. Probably, one of the reasons for such contradictory results could be attributed to evidence obtained from various animal models (i.e., type 1 or type 2 diabetes, and mice or rats) as well as from humans, because studies on non-wounded skin in diabetes are relatively fewer compared with those on other complications such as kidney, retina, and peripheral nerves.

The pathogenesis of skin complications in diabetes remains unclear. It is reported that diabetic neuropathy and microangiopathy are likely involved in the development of skin diseases, while a compromised immune system could account for increased susceptibility of the epidermis to infection [2]. Recently, Taylor et al found that the impaired function of gamma-delta (γδ) T cells could be a cause for reduced keratinocyte number and altered epidermal histology using two type 2 diabetes mouse models (db/db mice and high-fat diet-fed obese mice) [8]. While these mouse models are diabetic, the culprit for the keratinocyte abnormalities remains unclear as these mouse models exhibit several other metabolic abnormalities in addition to hyperglycemia, such as hyperlipidemia, obesity, and insulin resistance [8].

Here, we hypothesize that hyperglycemia *per se* induces the skin dysfunctions and disturbs keratinocyte homeostasis in mice. To test our hypothesis, skin barrier functions, morphological changes, and keratinocyte differentiation status were examined in the skin of streptozotocin (STZ)-induced diabetic mice, in which the main pathogenesis of diabetes was due to hyperglycemia. To dissect the effects of STZ toxicity, the effects of insulin were also assessed in this model.

## Materials and Methods

### Mice

The Animal Care Committee of Shiga University of Medical Science approved all experimental protocols (#2013-10-1H). Eight- to ten-week-old C57BL/6J mice were purchased from CLEA Inc. (Osaka, Japan). Hyperglycemia was induced by a single intravenous injection of STZ (150 mg/kg) (Nacalai Tesque, Kyoto, Japan) to establish an insulin-deficient diabetic mouse model. Mice injected with citrate buffer alone were used as control. For pellet implantation, mice were anesthetized by inhalation of 1.5% isoflurane (NARCOBIT-E, Natsume seisakusho Co, Ltd., Tokyo, Japan). STZ-injected mice were randomly divided in two groups to establish the following three groups in total: STZ-injected mice (n = 10) in which insulin was administered in the form of implanted microcrystallized palmitic acid pellets (LinBit, LinShin Canada, Inc., Canada), STZ-injected mice (n = 10) in which control pellets (Palmitic acid pellets, Innovative Research of America, Sarasota, FL) were implanted, and citrate buffer-injected mice (n = 10) in which control pellets were implanted. Both the insulin and control pellets were implanted subcutaneously in the back, followed by 5–0 nylon adaptation sutures. The mice were warmed up and monitored for several hours. All mice were checked twice a week and there was no unexpected death due to surgery. They were housed in a specific pathogen-free barrier facility and held under standard conditions in plastic cages (12 hours light: 12 hours dark photoperiod cycle; temperature 23 ± 2°C). Food and tap water were available *ad libitum*. Blood glucose levels were measured once a week in all experimental mice. The experiment was performed twice and the representative data were shown. To determine serum insulin concentration, mice were placed in a retainer to obtain 10 μl of blood by cutting tails. Serum was obtained after centrifugation at 3700 rpm for 30 minutes at 4°C the following day and kept at -80°C until insulin was measured. A Morinaga Ultra Sensitive Mouse/Rat Insulin ELISA kit (Morinaga Institute of Biological Science, Inc., Yokohama, Japan) was used to measure serum insulin concentration. All efforts were made to minimize pain due to surgery and to obtain reliable data from minimum number of experimental animals. Information on the number of animals used for each experiment is described in each figure legend.

### Skin barrier function tests

The dye penetration assay was performed according to Bognar et al. with modifications [14]. Briefly, mice were anesthetized by intraperitoneal injection of ketamine/xylazine/acepromazine (60 mg/kg, 10 mg/kg, and 2 mg/kg, respectively), followed by removing the hair of the ear skin using Nair (Church & Dwight Co., Inc. Ewing, NJ). 1 μl of 1 mM lucifer yellow (Sigma, St. Louis, MO) solution was applied onto the skin surface of the ears. After incubation for 3 minutes, the solution was removed and the ears were placed between a slide and a coverglass; the ears were photographed at 2.5μm intervals with a range of 120 to 165 μm using a laser-scanning confocal microscope to build Z-stack images (EZ-C1, Nikon, Tokyo, Japan). ImageJ software (NIH, Bethesda, MD) was used to evaluate stained areas and measure the diameters of leaked areas. Transepidermal water loss (TEWL) on the dorsal skin was measured using a Vaposcan (Asahi Techno Lab. Ltd., Kanagawa, Japan). Mice were anesthetized by inhalation of 1.5% isoflurane and hairs on the dorsal region (20 mm x 20 mm) were shaved with an electric clipper and then removed by Nair. On the following day, mice were anesthetized as in the previous day. After tape stripping four times using 3M Scotch mending tape (Sumitomo 3M Limited, Tokyo Japan), TEWL was measured five times in the same region per mouse and the average values were calculated.

### Histological examination

Mice were anesthetized with intraperitoneal injection as described in the previous section and exsanguination and perfusion with 4% paraformaldehyde were performed. Pieces of the dorsal skin were obtained and embedded in paraffin or Tissue-Tek OCT compound (Sakura Finetek Japan Co., Ltd., Tokyo, Japan). Sections (10 μm) were obtained for hematoxylin and eosin (H&E) staining and immunofluorescence. To perform immunofluorescence with epidermal sheets, ear skin was taken before the perfusion with 4% paraformaldehyde, split into dorsal and ventral halves, fixed with 95% ethanol on ice, and incubated in 3.8% ammonium thiocyanate for 30 minutes at 37°C. The epidermis separated from the dermis and was permeabilized in methanol at -20°C and then washed in 0.2% Triton-X PBS several times. Blocking was performed with 0.05% goat serum and 0.1% fetal bovine serum in 0.2% Triton-X PBS for 1 hour at room temperature, followed by incubation with ZO-1 (1:20, Cell Signaling Technology, Danvers, MA) for two consecutive nights at 4°C. Alexa 488 anti-rabbit IgG (1:400, Life Technologies, Grand Island, NY) was used as the secondary antibody. Nuclei were stained with TO-PRO-3iodide (Life Technologies). Epidermal sheets were photographed at 0.5 μm intervals with a range of 15 to 20 μm using a laser-scanning confocal microscope to build Z-stack images (EZ-C1). ImageJ software was used to evaluate stained areas and count epidermal cells. To evaluate epidermal cell density, epidermal sheets were prepared as in whole immunofluorescence with ZO-1 antibody. Epidermal sheets were fixed in 4% paraformaldehyde, stained with TO-PRO-3iodide and mounted with Vector Shield (Vector Laboratories, Burlingame, CA). At least 10 images per sample were acquired with a confocal microscope for calculating epidermal cell density. As for immunohistochemistry, paraffin or frozen sections were incubated with primary antibodies overnight at 4°C, followed by secondary antibodies for 2 hours at room temperature. Primary antibodies used were the following: anti-caspase 3 (1: 100, Cell Signaling Technology), anti-Ki67 (1:100, Abcam, Boston, MA), anti-keratin 5 (K5) (1:400, Lifespan, Seattle, WA or 1:500, Covance, Princeton, NJ), anti-keratin 14 (K14) (1:1000, Covance), anti-keratin 10 (K10) (1:500, Covance), anti-Filaggrin (1:1000, Covance), anti-Loricrin (1:500, Covance). Secondary antibodies used were ImmPRESS^™^ HRP Anti-Rabbit IgG detection kit followed by Vectastain ABC HRP kit (Vector Shield), Alexa Fluor 488 or Alexa Fluor 555 goat anti-rabbit or guinea pig IgG (1:1000, Life Technologies). Sections for immunofluorescence analyses were mounted with Vector Shield using 4´-6-diamidino-2-phenylindole (DAPI) and photographed with a laser-scanning confocal microscope. The number of caspase 3- or Ki67-positive cells and epidermal cells were quantified in each 3^rd^ section of the dorsal skin in the control and diabetic mice, respectively. Eight sections per mouse were examined.

### Cornified envelope (CE) isolation, quantitative analyses, and sonication experiments

CEs were prepared from 20 mm x 25 mm of ventral skin as previously described [15]. Briefly, CEs were isolated by boiling the extraction buffer (2% SDS, 100 mM Tris-HCl pH8.0, 5 mM EDTA, 20 mM DTT, followed by 0.2% SDS, 100 mM Tris-HCl pH8.0, 5 mM EDTA, 20 mM DTT) and by centrifuging (12, 000 x G) between each step, followed by resuspension in 1 ml of 100 mM Tris/EDTA. Next, 15 μl of CEs suspension was mounted and photographed using Q-IMAGING (British Columbia, Canada). At least 20 images per sample were acquired for counting CEs using ImageJ software. For the sonication experiments, CE concentration was determined with a hemocytometer and 1.0 x 10^4^ of CEs from the control, diabetic, and diabetic mice receiving insulin therapy was suspended in 2% SDS and adjusted to 60 μl. It was sonicated using a sonicator (THU-80, AS ONE, Tokyo, Japan) at level 1 for 20 minutes on ice. Then, 15 μl of CE suspension was mounted and photographed using Q-IMAGING. At least 6 images per sample were acquired in order to count destroyed and intact CEs using ImageJ software.

### Flow cytometry and Real-time PCR

The preparation of the epidermal cell suspension from ear skin and the cell sorting were performed as previously described [16]. Briefly, split ventral and dorsal skin of the ear was incubated in 0.5% dispase (Roche, Basle, Switzerland) for 45 minutes at 37°C. After separation of the epidermis from the dermis, epidermal sheets were incubated in 0.3% trypsin for 10 minutes at 37°C, followed by addition of an equal volume of RPMI (Wako Pure Chemical Industries, Ltd. Tokyo, Japan) with 10% fetal bovine serum and 0.1% DNase (Sigma). After vigorous shaking for 30 seconds, the cell suspension was filtered and collected after centrifugation at 1400 rpm for 10 minutes at 4°C. Amine reactive dye (LIVE/DEAD Fixable Violet Dead Cell Stain Kit (Life Technologies)) was added to exclude dead cells and then anti-mouse CD16/32 antibody (10 μg/ml) (clone 2.4G2, BD Pharmingen, Franklin Lakes, NJ) was used to block Fc-gamma (Fcγ) receptors before staining. Antibodies used for sorting included anti-CD45 (30-F11, BD Pharmingen), anti-MHCII (clone M5/114.15.2, eBioscience, San Diego, CA), anti-Sca-1 (D7, BD Pharmingen) and anti-Integrin α6 (GoH3, eBioscience). CD45^-^MHCII^-^ Sca-1^+^Integrin α6^+^ cells were sorted to collect basal cells, as previously reported [17]. To detect apoptotic cells, anti-annexin V antibody (BD Pharmingen) was used, and 1.0 x 10^6^ cells in epidermal cell suspension from control or diabetic mice were sorted to compare the percentage of amine reactive dye^+^ annexin V^+^ cells in CD45^-^MHCII^-^ cells. For RNA isolation, sorted cells were frozen in lysis buffer at -80°C. RNA was isolated using an RNeasy Mini Kit (QIAGEN, Valencia, CA). cDNA was synthesized using PrimeScript RT reagent kit (Takara, Kyoto, Japan) and real-time PCR was performed in triplicate using Light Cycler 480 SYBR Green I Master (Roche, Mannheim, Germany). To perform real-time PCR, the following primers were used: Keratin 5-F, TGATGACCTACATGAACAAGG; Keratin 5-R, AGACGTGTGTCTGCATCTGG; Keratin 14-F, CCTCTGGCTCTCAGTCATCC; Keratin 14-R, GAGCAGCATGTAGCAGCTTTAG; RPLP0-F, ATCAATGGGTACAAGCGCGTC; RPLP0-R, CAGATGGATCAGCCAGGAAGG. Individual gene expression was normalized against the ribosomal protein, large, P0 (RPLP0) housekeeping gene.

### Transmission electron microscope

Under inhalation anesthesia of 1.5 to 3% isoflurane, dorsal skin was obtained and fixed with 2% paraformaldehyde and 2% glutaraldehyde in 0.1 M cacodylate buffer, followed by 2% osmium tetroxide in 0.1 M cacodylate buffer. Samples embedded in polymerized resins were sectioned at 70 nm, mounted on copper grids, and observed using a transmission microscope (JEM-1400 Plus; JOEL Ltd., Tokyo, Japan) at an acceleration voltage of 80kV. Images were captured with a CCD camera (VELETA; Olympus Soft Imaging Solutions GmbH, Munster, Germany).

### Primary keratinocyte culture and cell proliferation assay

**Fig 1:**
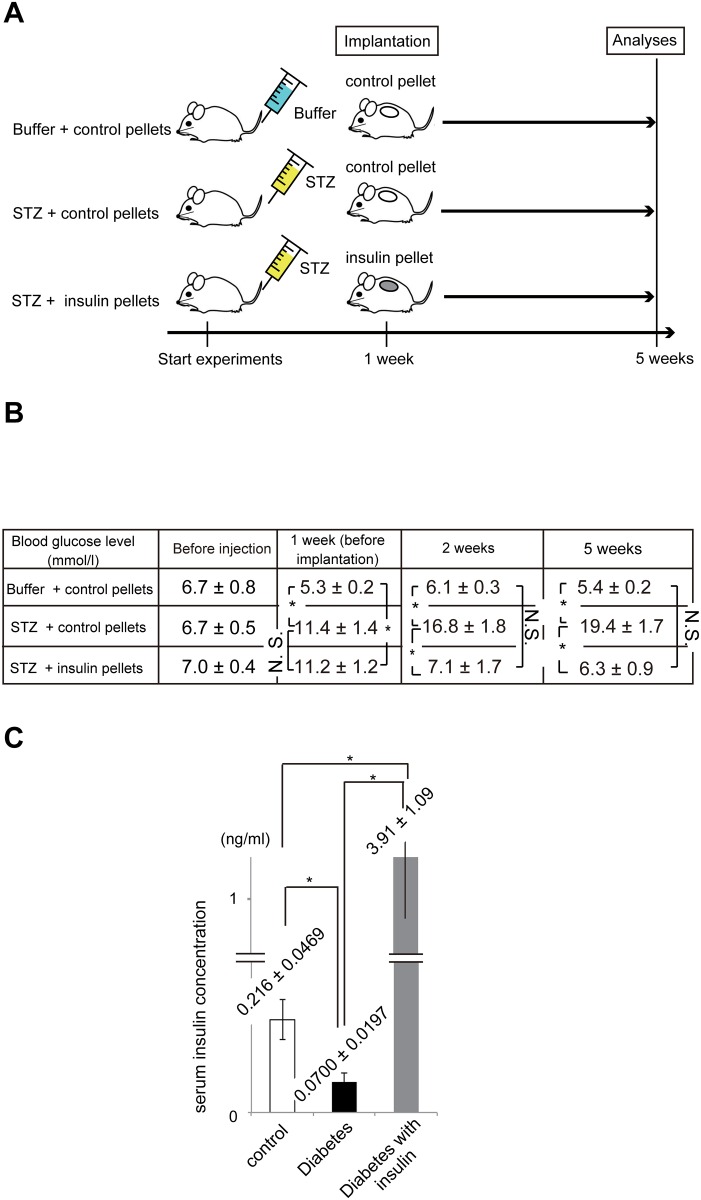
Time course and physiological conditions of mice. (A) Schematic illustration of mouse models. (B) Blood glucose levels (mmol/l) before STZ or buffer alone injection, at 1 week, 2 weeks, and 5 weeks after the injection are shown for the three groups. Before STZ or buffer alone injection, there was no significant difference in blood glucose levels among the three groups. (C) Serum insulin concentration (ng/ml) is shown at 5 weeks after the injection in the three groups. The average values ± standard errors (S. E.) of 10 mice in each group are shown. N.S., not significant; *P < 0.01.

Neonate skin was unwrapped from the body using forceps after CO_2_ exposure and decapitation [18]. The unwrapped neonate skin was incubated in 0.5% dispase overnight at 4°C. On the following day, the epidermis was separated from the dermis and incubated into TrypLE (Invitrogen, Carlsbad, CA) for 10 minutes with shaking at 37°C. Then, CnT-PR (CELLnTEC, Bern, Switzerland) was added and pipetted 20 times to create the epidermal cell suspension. After filtration and centrifugation at 200 g for 10 minutes, 4 x 10^4^ cells were plated per well in rat-tail collagen (Sigma) -coated 48 well plates. The following condition of medium was examined (5 wells per group): CnT-PR with 38 mM of glucose, CnT-PR with 7.5 mM of STZ, which corresponded to the concentration in blood when 150 mg/kg of STZ was injected in a mouse, CnT-PR with 4.0 ng/ml of insulin (Wako Pure Chemical Industries), which corresponded to the average concentration of insulin in blood of the diabetic mice with insulin treatment (Fig 1C), and CnT-PR (control). The setting of high glucose concentration (38 mM in this study) followed the protocol in a previous study in which the physiological high glucose concentration was discussed and *in vitro* experiments performed [19]. To dissolve STZ or insulin, saline was used and the same amount of saline was added to the control. 1% Antibiotic-Antimycotic (Thermo Fisher Scientific, Waltham, MA) was routinely added in CnT-PR. Glucose concentration in Cn-TR was 8 mM, which is thought to be the normal glucose level [20]. After 24 hours cultivation, WST-1 (Takara, Shiga, Japan) was added according to the instruction manual and the absorbance at 440 nm was measured using a multi-well plate reader (Infinite F200, TACAN, Kawasaki, Japan). The absorbance at 650 nm was used as the reference wavelength. Experiments were performed twice and representative data are shown.

### Statistical analysis

One-way ANOVA followed by Bonferroni correction was used to assess the significance of the data. *P < 0*.*01* was considered statistically significant.

## Results

### Diabetic mouse models with/without insulin pellets

Diabetes was induced in C57Bl/6 mice by intravenous administration of STZ. Either control or insulin pellets were subcutaneously placed under the mid dorsal skin in half of the diabetic mice (Fig 1A). Compared to age-matched control mice in which control pellets were implanted (Fig 1A), the diabetic mice showed significantly higher blood glucose concentration at 1 week, 2 weeks, and 5 weeks (Fig 1B). In turn, insulin therapy significantly increased serum insulin levels and lowered blood glucose concentrations in the diabetic mice (Fig 1B and 1C).

### Hyperglycemia disturbs skin barrier function in the diabetic mice

**Fig 2:**
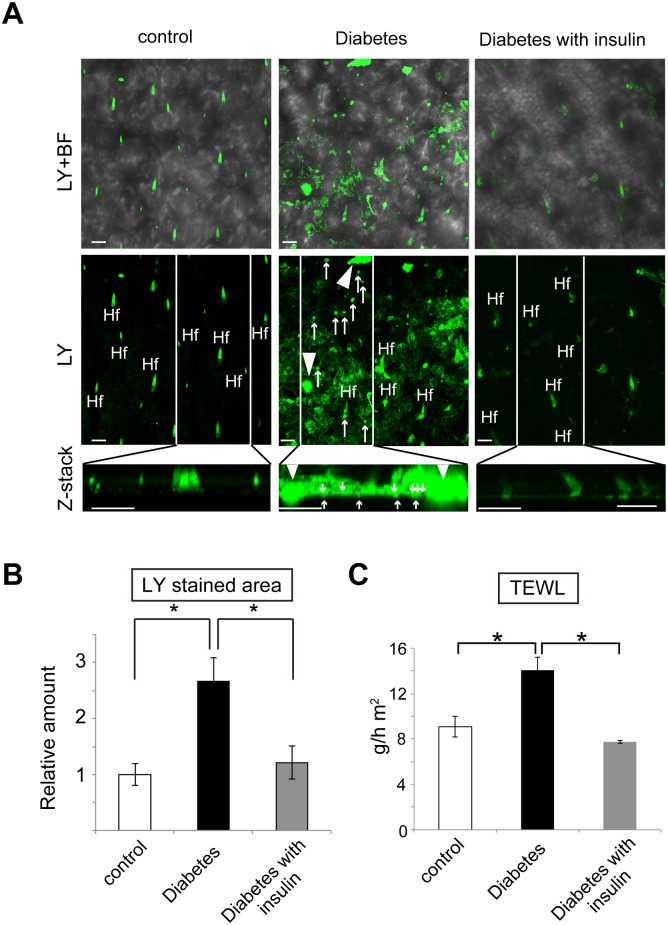
Skin barrier dysfunction in diabetic mice and its mitigation by insulin treatment. (A) *In vivo* lucifer yellow (LY) penetration assay reveals dye leakages in the stratum corneum of the diabetic mice. Arrowheads indicate large leakages, while arrows indicate small leakages. Hf, hair follicles. Top panels (LY + bright filed (BF)) show the merged images of dark (middle panels) and BF in the epidermis. Bottom panels (“Z-stack”) are the magnified images of a 90° rotation of the white boxes in middle panels. Scale bars = 50 μm. Four mice per group were examined. (B) LY stained areas are shown in the skin from the control (white bar), diabetic mice (black bar) and insulin-treated diabetic mice (gray bar). Values are relative to control (set as 1) ± S.E. Four mice per group were examined. (C) Transepidermal water loss (TEWL) after tape stripping is shown for the control mice (white bar), the diabetic mice (black bar), and insulin-treated diabetic mice (gray bar). The average values ± S. E. of 10 mice in each group were shown. Experiments shown in (A), (B), and (C) were repeated twice. *P < 0.01.

Skin functions as a barrier to prevent the invasion of external assaults such as chemical materials and microorganisms from the outside (outside-in skin barrier). Lucifer yellow (LY) dye with 0.95 nm in diameter cannot penetrate into the epidermis [21], therefore it was utilized to examine the epidermal barrier functions against external assaults. In particular, the outside-in barrier function in the stratum corneum would be an important target to evaluate [15, 22]; therefore, the recently developed *in vivo* dye penetration assay [14] was used in this study. Consistent with previous studies [15, 22], we found that positive signals were confined to hair follicles and to the outer surface of the stratum corneum in the control mice (Fig 2A). By contrast, LY signals were sporadically observed in the stratum corneum of the diabetic mice (Fig 2A). Quantification of LY positive signals showed that total LY stained area significantly increased by 2.7 ± 0.4-fold (mean ± standard errors) in the diabetic mice, compared to the control mice (Fig 2B). These data indicate that the outside-in barrier was impaired in the diabetic mice. Importantly, controlling the blood glucose with insulin in the diabetic mice returned the total LY stained area to a similar amount as that of the control (Fig 2B). Thus, the impaired outside-in barrier would be caused by hyperglycemia, but not by STZ toxicity.

Another barrier function of the skin is to regulate water evaporation from the epidermis (inside-out skin barrier). TEWL is thought to be a marker for water evaporation from inside the body to the outside via the epidermis [23]. TEWL is also associated with water diffusion in intercellular spaces [24]. In this study, a tape stripping technique was applied prior to TEWL measurements because this technique adequately removes corneocytes to evaluate the function of the epidermis [25]. It was found that TEWL significantly increased in the diabetic mice compared to the control mice (Fig 2C). Importantly, insulin treatment prevented a TEWL elevation observed in the diabetic mice (Fig 2C).

### Hyperglycemia changes the distribution of tight junction protein-1 (ZO-1)

**Fig 3:**
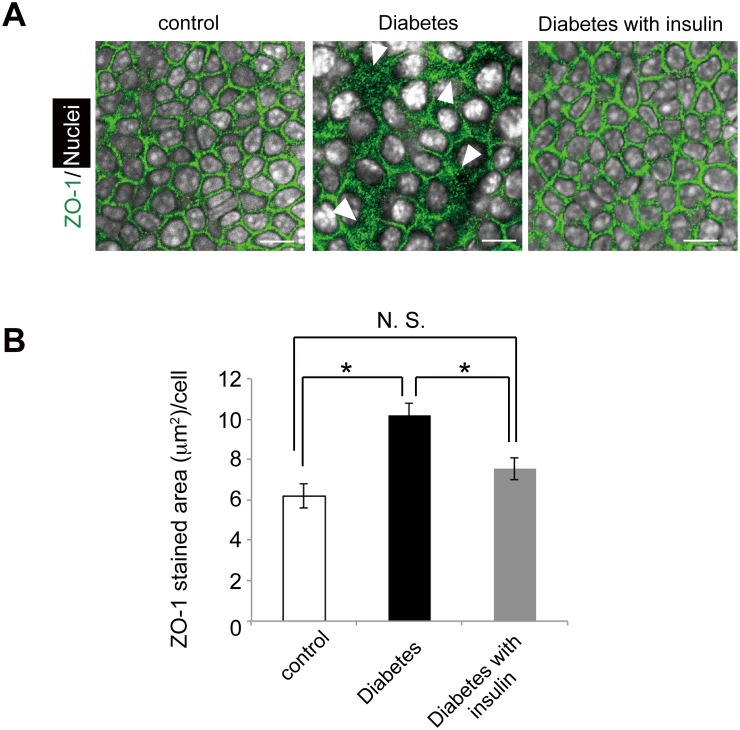
Different distribution of tight junction protein expression in the diabetic mice and the effect of insulin treatment. (A) ZO-1 (green) expresses widely and diffusely in the skin of diabetic mice among epidermal cells. The nuclei of epidermal cells are indicated in white color. Arrowheads indicate significantly widened area with ZO-1 positive in the diabetic mice. Scale bars = 10 μm. (B) ZO-1 stained area per epidermal cell (mean ± S.E.) is shown in three groups. Four mice per each group were examined and experiments were repeated twice. *P < 0.01.

Cell-to-cell junction in the granular layers of the skin is sealed by tight junctions, which enables the regulation of water evaporation from the body as well as block external assaults [26, 27]. Since both the outside-in and the inside-out skin barrier did not function normally in the diabetic mice, we next investigated whether hyperglycemia could alter the expressions of tight junction proteins between keratinocytes. It was found that tight junction protein-1 (ZO-1) was more widely and diffusely expressed among the epidermal cells in the diabetic mice than in the control mice (Fig 3A). A quantitative analysis confirmed that ZO-1 positive area per keratinocyte was significantly greater in the diabetic mice compared with that in the control mice (Fig 3B). Importantly, such enhancements were mitigated by insulin treatment in the diabetic mice (Fig 3A and 3B). Given these facts, the altered distribution pattern of the protein associated with the tight junction among keratinocytes might account for the impairment of the skin barrier function in the diabetic mice.

### Diabetes alters epidermis histology in the mice

**Fig 4:**
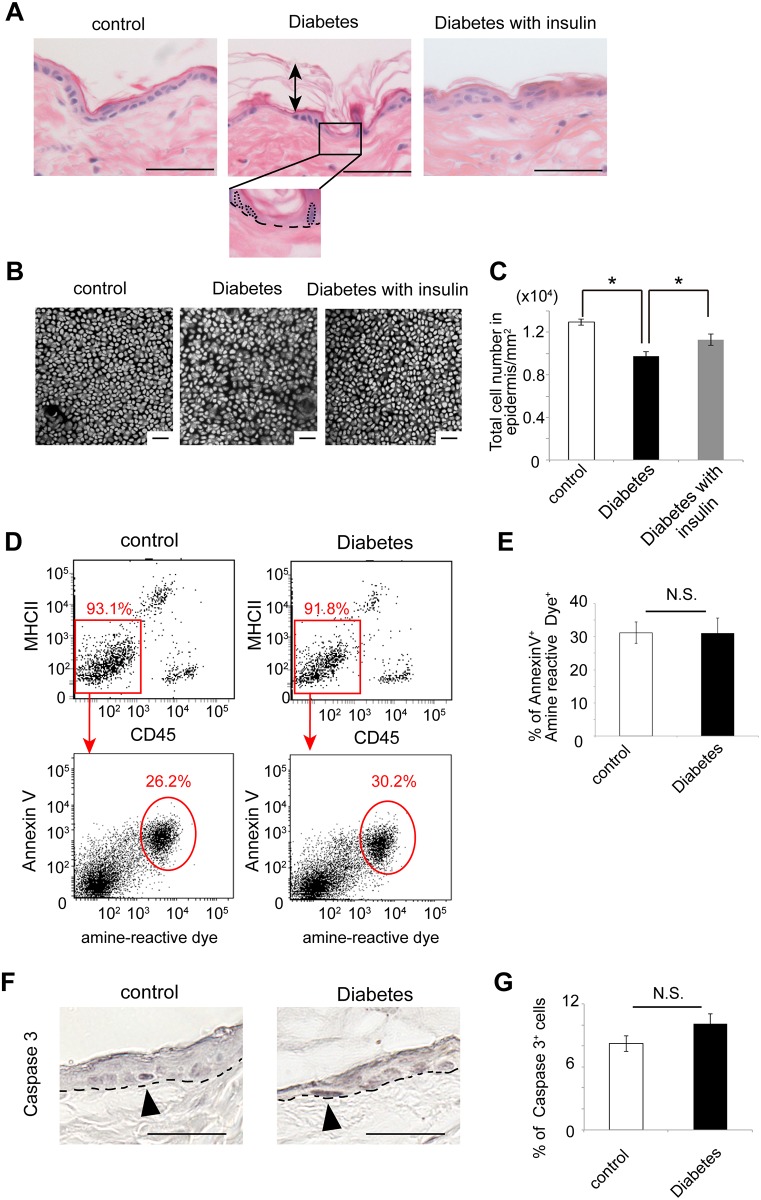
Altered skin morphology but no significant induction of apoptotic cells in the epidermis of the diabetic mice. (A) The H&E staining showed the alignment of basal cells in the dorsal skin of the control and insulin-treated diabetic mice, but the alignment was lost in the diabetic mice. A black box in diabetes is magnified in the bottom panel and dot circles indicate nuclei of basal cells. Dot lines indicate the epidermis-dermis junction, on which basal cells are located. Arrows indicate the widened stratum corneum in the diabetic mice. (B) The nuclei staining in the epidermal sheets prepared from the ear skin are shown. (C) Epidermal cell numbers per 1 mm^2^ in the control (white bar), the diabetic mice (black bar) and insulin-treated diabetic mice (gray bar) were quantified. (D) Representative flow cytometry plots show that 93.1% and 91.8% of cells isolated from the enzymatically digested epidermal sheets of the control mice and diabetic mice, respectively, are CD45^-^MHCII^-^ cells. Next, 26.2% and 30.2% of epidermal cells turn positive for Annexin V and amine-reactive dye, which corresponds to apoptotic cells. Values adjacent to the red boxes or circles are the percent cells in each area. (E) Annexin V^+^ amine-reactive dye^+^ population in the epidermal cells (mean ± S.E.) between control (white bar) and diabetic mice (black bar). (F) Apoptotic cells in the epidermis were examined using anti-caspase 3 antibody by immunohistochemistry. Arrowheads indicate caspase 3-positive cells. Dot lines indicate the epidermis-dermis junction. (G) Caspase 3^+^ population in the epidermis cells (mean ± S.E.) between control (white bar) and diabetic mice (black bar). Scale bars = 10 μm in (A), (B) and (F) N.S., not significant; *P < 0.01. Five mice per each group for (A, F and G), four mice per each group for (B, C), and three mice per each group for (D, E,) were examined and experiments were repeated twice.

Histological analyses revealed that basal cells were regularly aligned in the basal layer of the control mice, while their alignment appeared irregular in the diabetic mice (Fig 4A). In addition, increased layers of the stratum corneum were also found in the diabetic mice (Fig 4A). The former finding seemed to be accompanied by decreased number of keratinocytes. Thus, to address the question whether keratinocytes were reduced in non-wounded skin of diabetic mice, nuclear staining was performed on the epidermal sheets obtained from ear skin. Consequently, the epidermal cell number was significantly reduced in the diabetic mice compared to that in the control mice (Fig 4B and 4C), which is consistent with the results from a recent report on the skin phenotype of type 2 diabetes [8].

In order to identify a mechanism for the decreased number of keratinocytes, we examined if keratinocytes underwent apoptosis in diabetic mice. By sorting for CD45^-^MHCII^-^ cells, we were able to isolate the population which was mostly composed of keratinocytes from the epidermis [17, 28]. Then, we examined if isolated epidermal cells contained apoptotic cells by labeling them with Annexin V and amine-reactive dye. It was found that the number of apoptotic cells in the diabetic mice were not different from that in the control mice (30.2% in diabetic mice vs. 26.2% in control mice, P = non-significant.) (Fig 4D and 4E). Simultaneously, immunohistochemical analysis using anti-caspase 3 antibody to detect apoptotic cells revealed that there was no significant difference of the percentage of apoptotic cells in the epidermis between the control and the diabetic mice (8.23% in diabetic mice vs. 10.1% in control mice, P = non-significant.) (Fig 4F and 4G).

**Fig 5:**
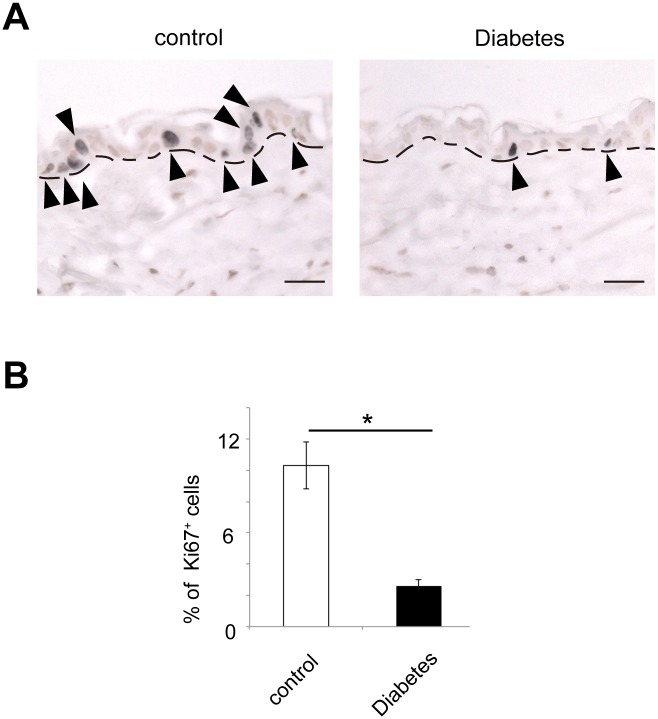
Reduced proliferation in the epidermal cells of the diabetic mice. (A) Ki67-positive cells (arrowheads) were significantly reduced in the epidermis of the diabetic mice compared with the control mice. Dot lines indicate the epidermis-dermis junction. Scale bars = 50 μm. (B) Ki67^+^ population in the epidermis cells (mean ± S.E.) between control (white bar) and diabetic mice (black bar). *P < 0.01. Five mice per group were examined.

In turn, we asked whether hyperglycemia affected proliferation of epidermal cells. Ki67-positive cells was significantly decreased in the epidermis of diabetic mice, compared with that of control mice (Fig 5A and 5B), which was consistent with a different approach by other group [6].

**Fig 6:**
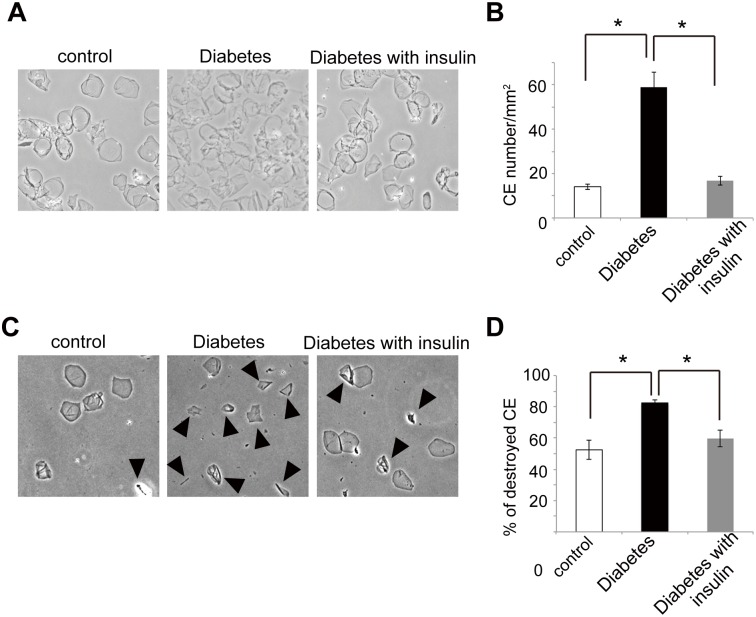
Aberrant cornified envelopes and their recovery by insulin treatment in the diabetic mice. (A) The cornified envelopes (CEs) were isolated from the same area of dorsal skin in the three groups. (B) Quantification of CE numbers per 1 mm^2^ (mean ± S.E.) in the skin of the control (white bar), diabetic mice (black bar) and insulin-treated diabetic mice (gray bar) is shown. (C) CEs isolated from control, diabetic, and insulin-treated diabetic mice are shown after 20 minute sonication. (D) Percentage of destroyed CEs (mean ± S.E.) after 20 minutes sonication in control (white bar), diabetic mice (black bar) and insulin-treated diabetic mice (gray bar) is shown. Arrowheads indicate destroyed CEs. *P < 0.01. Three mice per each group for (A-D) were examined and experiments were repeated twice.

Corneocytes are denucleated and terminally-differentiated keratinocytes located in the stratum corneum. These cells use CEs as scaffold instead of plasma membrane [29]. Here, we found an unbalanced association between the number of epidermal nucleated cells and CEs. In fact, while the number of epidermal cells decreased (Fig 4B and 4C), the number of CEs was rather higher in the diabetic mice than in the control mice (Fig 6A and 6B). Importantly, these changes observed in the diabetic mice were prevented by insulin treatment (Figs 4A–4C, 6A and 6B). Next, we addressed whether these increased CEs would be more vulnerable against mechanical stress by sonication in diabetic mice [30]. It was found that sonication destroyed 82.4 ± 1.94% (mean ± standard errors) of CEs derived from diabetes whereas 52.4 ± 6.08% of CEs of control mice were destroyed (Fig 6C and 6D). Again, insulin treatment was able to significantly reduce the number of fragile CEs in diabetic mice, because the destroyed CEs by sonication were 59.5 ± 5.25% in diabetic mice treated by insulin (Fig 6C and 6D). These results suggest that the integrity of CEs would be impaired despite of the significant increase of CE numbers in diabetic mice. Considering that basal cells physiologically move up to the suprabasal layers to finally differentiate into corneocytes, hyperglycemia would disrupt the differentiation process in the skin of the diabetic mice.

### Diabetes alters the ultrastructure in basal cells

**Fig 7:**
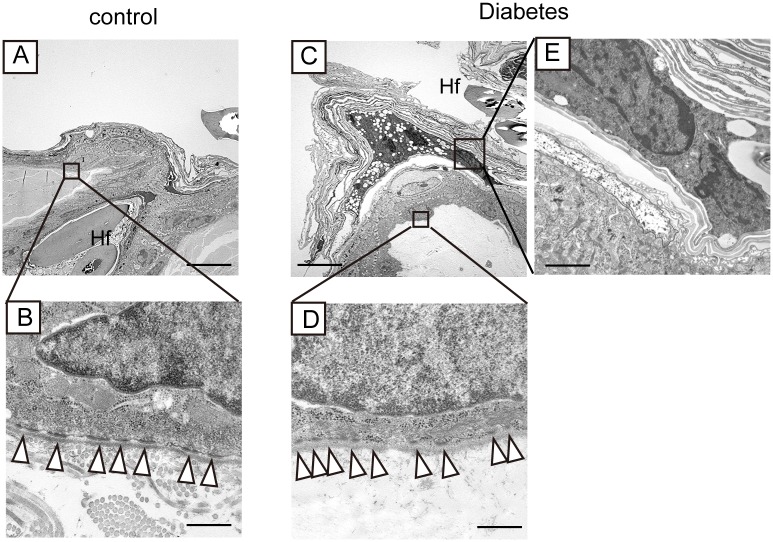
Ultrastructural analyses of non-wounded skin from the diabetic mice. Low magnification of the skin of control and diabetic mice is shown in (A) and (C). Black boxes are magnified in (B, D, and E). (B, D) Hemidesmosomes are indicated by white arrowheads. (E) Parakeratotic nuclei are present in the stratum corneum of the diabetic mice. Hf, hair follicle. Scale bars = 20 μm in (A, C), 2.5 μm in (E), and 500 nm (B, D). Two mice were examined in the control and the diabetic mice, respectively.

We next investigated the skin histology in more detail at the ultrastructural level. Transmission electron microscopy revealed that the skin of diabetic mice exhibited unclear and shorter hemidesmosomes in basal cells (Fig 7A, 7B, 7C and 7D). Some suprabasal cells exhibited abnormal parakeratotic nuclei in the stratum corneum in the diabetic mice (Fig 7C and 7E). Since it was reported that epidermal parakeratotic nuclei were associated with impaired barrier function of the skin [31, 32], this finding also supported the findings on the skin barrier defects in the diabetic mice.

### Epidermal differentiation is disturbed in the diabetic mice

The epidermal differentiation process is regulated in physiological conditions. Conversely, it is not fully determined as to whether hyperglycemia alters the physiological epidermal differentiation process [13]. Here, we examined several markers to identify the epidermal differentiation status in these mice.

**Fig 8:**
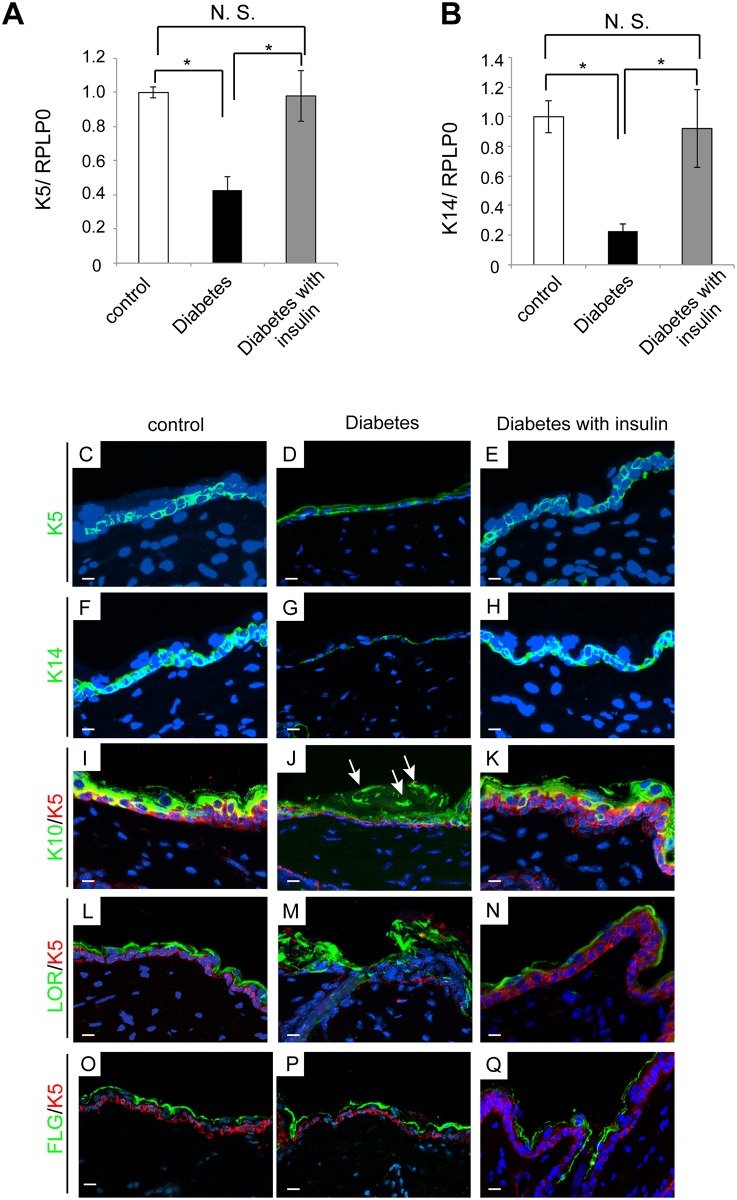
Aberrant expression of skin proliferation/differentiation markers in the diabetic mice. (A, B) *Keratin 5 (K5)* and *14 (K14)* expression was determined by real-time PCR using cDNA prepared from CD45^-^MHCII^-^ Sca-1^+^ Integrin α6^+^ cells. Individual gene expression was normalized to that of the ribosomal protein, large, P0 (RPLP0) housekeeping gene. Four mice were examined in each group and experiments were repeated twice. Representative data are shown. (C-Q) The immunofluorescence analysis of the skin sections is presented. Green signals indicate K5 (C-E) or K14 (F-H) in basal layer. While keratin 10 (K10, green) is positive in the suprabasal cells in control mice (I), it was also positive in the stratum corneum in diabetic mice (arrows in J). Likewise, loricrin (LOR) was also present in the stratum corneum as well as the granular and spinous layers (M), while it was detected in the granular and spinous layers of control mice (L). Filaggrin (FLG) expression (green in O-Q) was not dramatically changed. Insulin therapy dramatically inhibited these alterations in diabetic mice (E, H, K, N, Q). Nuclei are stained with DAPI (blue). Scale bars = 10 μm. Five mice were examined in each group and experiments were repeated twice.

First of all, basal cells (CD45^-^ MHCII^-^ Sca-1^+^ Integrin α6^+^) were isolated from the epidermal sheets by a cell sorter, and then the expressions of both *keratin 5* (*K5*) and *keratin 14* (*K14*) mRNAs were examined. It was found that these factors were significantly reduced in the diabetic mice (Fig 8A and 8B). Consistent with these findings, immunohistochemistry confirmed that the expressions of both K5 and K14 markedly decreased in the diabetic mice (Fig 8C–8H). Because K5 and K14 are markers of proliferation [33], this result suggests hyperglycemia led to less proliferation in the epidermis. In turn, the expression pattern for Keratin 10 (K10) and loricrin appeared different between the control mice and the diabetic mice. While these two proteins were physiologically confined to suprabasal cells in the control mice, they were ectopically positive in the stratum corneum of the diabetic mice (Fig 8I, 8J, 8L and 8M). Finally, the expression of filaggrin in the diabetic mice was likely identical to that in the control mice (Fig 8O and 8P). Importantly, these altered expressions of the keratinocyte differentiation markers were blocked by insulin treatment (Fig 8A–8Q). Altogether, these data suggest that hyperglycemia could perturb epidermal differentiation.

### Hyperglycemia, but not STZ, is responsible for skin disease in the diabetic mice

**Fig 9:**
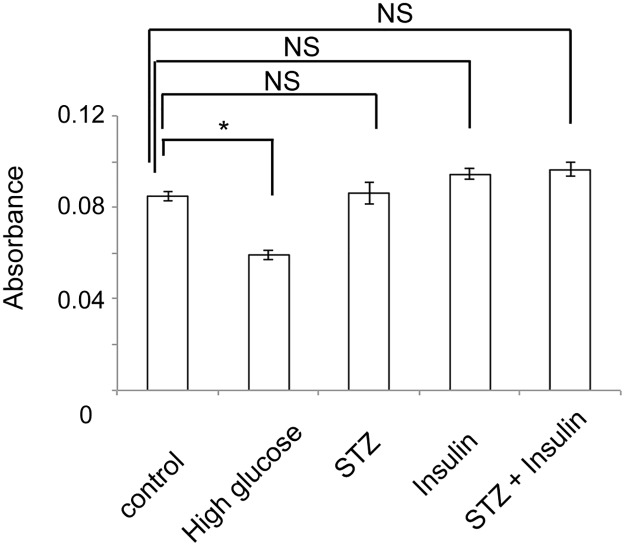
High glucose, but not STZ, insulin, or a combination of STZ and insulin, suppressed proliferation of keratinocytes *in vitro*. Different media that corresponded to the conditions in vivo in our model were examined to check whether these media conditions affected proliferation of keratinocytes. Experiments were repeated twice. *P < 0.01.

An important issue to be addressed in our animal model is the cytotoxicity of STZ. Hyperinsulinemia due to insulin pellets would be also another potential factor that affects keratinocyte homeostasis. Though we established another mouse model, control mice with insulin pellets, all died because of severe hypoglycemia before analyses. For these reasons, the effects of STZ and insulin on keratinocyte proliferation were examined in primary mouse keratinocyte cultures. Consistent with previous reports [7, 34] and our data *in vivo* (Fig 5), hyperglycemia reduced cell proliferation (Fig 9). Conversely, STZ, insulin, or a combination of STZ with insulin had no effect on keratinocyte proliferation (Fig 9). These data suggest that neither STZ nor insulin may have any major effects on keratinocyte proliferation.

## Discussion

In this study, we demonstrated that the skin barrier dysfunction with impaired the proliferation/differentiation processes of keratinocytes in diabetic mice was attributable to hyperglycemia.

It has been reported that skin complications develop in 11.4% to 71% of diabetic patients [13, 33]. In particular, skin infections are often deteriorating in diabetic patients, resulting in higher mortality and long-term hospitalization [5, 35–38]. While precise mechanisms remain unknown, these skin diseases would be associated with increase in the susceptibility to microbial infection in diabetes [39, 40]. Since skin physiologically has the outside-in barrier that blocks the invasion of harmful substances and pathogenic microorganisms from the outside into the epidermis, the susceptibility to microbial infection in diabetes might be associated with the skin barrier dysfunction.

Here, we examined the LY dye leakage to assess the outside-in barrier function in the stratum corneum of diabetic mice. This method allowed us to determine spots where the skin barrier was damaged. We found that several LY dye spots were larger than 10 μm in diameter (S1 Fig). Conversely, fungal infections have been statistically more common in diabetic patients, compared to the control group and of note, skin and nail mycosis often leads to limb/toe amputation in diabetic patients [39]. Given the fact that the size of the hypha of fungi is approximately 6–12 μm in diameter [41], our data suggest that such barrier defects in the non-wounded skin in diabetes may allow hypha of fungi to easily invade into the stratum corneum.

Another skin function is the inside-out skin barrier, through which water evaporation from epidermis is adequately regulated in order to maintain biological body fluid homeostasis. Xerosis is an example of skin disease where such barrier functions are impaired [42]. In fact, the skin disease is characterized by dry skin and persistent itching, and is commonly observed in diabetic patients [13]. For these reasons, it is likely that the inside-out barrier function would be impaired in diabetic patients. Given the hypothesis, TEWL has been utilized to evaluate the barrier function in diabetic patients as well as in experimental animals [37–40]. However, these studies showed that TEWL was not altered in diabetes compared with the control [6, 9, 43, 44]. This result was not in agreement with the hypothesis and therefore, Quondamatteo pointed out the possibility that altered structures such as cutaneous microcirculation might mask the alteration of TEWL or that barrier defect signs other than TEWL might exist in diabetic patients and animal models [13].

Tape stripping is an efficient method to remove corneocytes [45]. In general, tape stripping is utilized to examine stratum corneum mass, barrier function and penetration of topical substances [46]. Another application of tape stripping is to reveal existing barrier defects. For instance, Ackerl et al. demonstrated that tape stripping revealed a significant increase of TEWL in plectin-conditional knockout mice compared with that in the control group, although TEWL was comparable between these knockout and control mice before tape stripping [25]. Based on our finding of abundant corneocytes in non-wounded skin of diabetes, we hypothesized that a significant number of corneocytes might mask an abnormal inside-out barrier in the skin of diabetic mice. Hence, a tape stripping prior to TEWL measurement revealed that TEWL significantly increased in the diabetic mice compared to that in the control mice. Basically, tape stripping usually causes inflammation due to its mechanical stress [25]. Indeed, more than 12 tape strips were reported to induce inflammation, resulting in a significant increase in TEWL [47, 48]. We confirmed such findings in our model (S2 Fig). However, a problem is that the cause of an increased skin permeability remains unclear. For these reasons, mild way of tape stripping (4 tape strips) was applied in this study [25, 49] to impair only skin barrier function without inflammation. Altogether, our data suggest that the skin of diabetic mice is more susceptible to the inside-out barrier defects with the onset of external stimulus, compared with the skin of control mice.

In physiological epidermis, keratinocytes express several markers upon movement into the suprabasal layers. The final step is the conversion of living cells into corneocytes, which are scaffolded by cell envelopes. Importantly, the proliferation/differentiation process is tightly controlled in physiological conditions [50]. Our data showing a decreased expression of K5 and K14 in the basal cells, an ectopic expression of K10/loricrin, and sensitivity of cell envelopes in the diabetic mice to mechanical stress, all of which would suggest that hyperglycemia disrupted the proliferation/differentiation process of keratinocytes in the diabetic mice.

Since we demonstrated that ZO-1 distribution in the epidermis was associated with skin barrier dysfunction by experiments using diabetic mice and those with insulin treatment, altered ZO-1 distribution appears to contribute to skin barrier dysfunction. We showed that ZO-1 positive area per keratinocyte was increased in type 1 diabetic mice, while Taylor et al reported greater area with adherens junction protein, E-cadherin positive per keratinocyte [8]. Intriguingly, it has been shown that ZO-1 regulates barrier formation and adherens junctions through VE-cadherin in endotheial cells [51]. Additionally, hyperglycemia alters ZO-1 distribution through the down-regulation of Cx43, gap junction protein, leading to barrier disruption in airway epithelium [52]. In contrast, the precise mechanism of the process of the alteration of ZO-1 expression to cause barrier dysfunction is still unclear in the skin of diabetic mice. Future study to examine the role of ZO-1 in the epidermis of diabetes should be warranted.

It is unlikely that STZ would be a cause for keratinocyte alterations as STZ did not exhibit cytotoxicity of keratinocytes. In addition, skin phenotype in STZ-injected mice was ameliorated by insulin treatment. However, there are a couple of studies mentioning STZ could be cytotoxic to keratinocytes [53]. Such difference could be attributed to differing concentrations of STZ used and different cell types. 10 mM STZ was used on HaCat cells in other studies, whereas 7.5 mM was administered to primary keratinocytes in this study. Since systemic administration of 150 mg/kg STZ would correspond to a blood concentration of 7.5 mM, we believe that application of 7.5 mM of STZ to primary keratinocytes would be suitable. Perhaps, the safety window for STZ usage might be very narrow and 10 mM STZ might be potent enough to affect keratinocyte function.

Finally, we tested the effect of insulin on cultured keratinocytes. Since insulin has multiple protective actions on several organs in addition to lowering blood glucose [54], it is safe to assume that the direct effect of insulin might contribute to ameliorating skin damage. We found that insulin did not have any effect on keratinocyte proliferation, suggesting that the protective effects of insulin were attributed to controlling blood glucose concentration, but not to the direct effects from insulin *per se*.

Overall, our study directly demonstrated that both the outside-in barrier and the inside-out barrier are impaired by hyperglycemia with alterations of keratinocyte integrity in type 1 diabetic mice. Our findings would be helpful for the understanding of the mechanisms behind the development of skin complications in diabetes.

## Supporting Information

S1 Fig The rates of occurrence of arbitrarily sized dye leakage spots in the stratum corneum of diabetic mice. Dye leakage spots were counted according to the size of diameter from examined four diabetic mice. Experiments were repeated twice and representative data are shown. Both control and insulin-treated diabetic mice had no dye leakage.
(EPS)

S2 Fig TEWL after twelve tape strips in control, the diabetic, and insulin-treated diabetic mice. There was no significant difference in the three groups. The average values ± S. E. of 10 mice in each group were shown. Experiments were repeated twice.
(EPS)
